# Dataset for common wheat (*Triticum aestivum* L.) grain and flour characterization using classical and advanced analyses

**DOI:** 10.1016/j.dib.2025.111375

**Published:** 2025-02-07

**Authors:** Mélanie Munch, Laura Rezette, Patrice Buche, Baptiste Chambrey, Catherine Deborde, Stéphane Dervaux, Sonia Geoffroy, Kamal Kansou, Sophie Le Gall, Laurent Linossier, Benoit Meleard, Luc Menut, Marie-Hélène Morel, Magalie Weber, Luc Saulnier

**Affiliations:** a INRAE, UMR 1253 STLO, Institut Agro Rennes, 35042 Rennes, France; b INRAE, UR 1268 BIA, 44300 Nantes, France; c INRAE, PROBE research infrastructure, BIBS Facility, 44300 Nantes, France; d INRAE, UMR 1208 IATE, University of Montpellier, Institut Agro Montpellier, 34060 Montpellier, France; e Université Paris-Saclay, INRAE, AgroParisTech, UMR 518 MIA Paris-Saclay, 91120 Palaiseau, France; f Arvalis-Institut du Végétal, Station Expérimentale, Boigneville 91720, France; g Limagrain Ingredients, ZAC Les Portes de Riom, Avenue George Gershwin, Riom 63200, France; h Axiane Meunerie, Val d'Anast 35330, France

**Keywords:** Bread, Wheat, Grain quality, Biochemical composition

## Abstract

As global warming and changing market demand reshape agricultural practices, optimising the quality and utility of crop products, particularly wheat, is becoming increasingly complex and critical. Wheat plays a central role in human and animal nutrition, with its quality influenced by multiple factors at different scales, from grain composition to end-product performance, usually evaluated through sensory evaluation. Understanding the relationship between wheat composition and technological quality is essential for improving product value in agri-food systems. This dataset represents a broad panel of wheat samples encompassing diverse genetic backgrounds grown under varying environmental conditions in France. It collects measurements of grain, flour, dough and bread characteristics, facilitating a comprehensive comparison of wheat quality at different stages of production. The dataset encompasses 35 classical technological tests, 31 detailed compositional analyses—including in-depth characterization of protein composition (glutenin and gliadin), pentosan content measurement, and fatty acid profile analysis—and 37 sensory evaluations from the French Bread baking test providing detailed assessments of flour quality and dough behavior across key bread-making stages. In addition, raw data sets from Alveograph® and Farinograph® tests are included to support the development of innovative quality assessment criteria. This dataset will be valuable not only for the crop industry in its efforts to optimize wheat quality, but also for researchers and data scientists exploring the complex relationships between composition, processing and final bread quality. The data are registered in the French Research Data Gouv public repository and also stored in the PO2 Evagrain database using the PO2/TransformON ontology. The SPO2Q web tool allows for online database consultation, with further access available through the PO2 Manager desktop application.

Specifications Table

**Table:** 

Subject	Agronomy and Crop Science
Specific subject area	Cereal Science
Type of data	Table Raw, Processed, Analysed
Data collection	**Samples:** 290 wheat samples harvested in 2020 (27), 2021 (123) and 2022 (140), including 99 different varieties and 39 growing locations across France (38) and Belgium (1). **Milling:** Flours were obtained using an experimental mill **(**MCKA, Bülher, Switzerland)
	**Wheat Grain characterization:** Water and Protein composition (near-infrared spectroscopy NIRS – NF EN 15948), Hardness (NIRS – calibration with Particle Size Index), Specific weight (NF EN ISO 7971-3), Thousand kernel weight (NF EN ISO 520), Hagberg Falling Number (Falling number 1500 – ISO 3093:2009), Gluten Index (Glutomatic – NF EN 21415-2). **Wheat Flour characterization:** Alveogram (CHOPIN Alveograph – NF EN ISO 27971), Farinogram (CHOPIN Mixolab – NF V03-765), Polysaccharide and Lipid composition (GC-FID), Protein composition (SE-HPLC), **Wheat Flour and bread characterization:** Bread Baking Test (NF V03-716)
Data source location	**Limagrain Ingredients**, ZAC Les Portes de Riom, Avenue George Gershwin, Riom FR-63200 **Axiane meunerie**, Val d'Anast, FR-35330 **Arvalis**, Station Expérimentale, Boigneville FR-91720 **INRAE** in - BIA-BIBS Facility 1268 Biopolymères Interactions Assemblages, Nantes FR-44316, IBiSA, Phenome-Emphasis-FR ANR-11-INBS- 0012, CALIS/ PROBE research infrastructures, Biogenouest (10.15454/1.5572358121569739E12) - PLANET-IATE, 1208 Agropolymers and Emerging Technologies Facility, Montpellier FR-34060, CALIS research infrastructure, (10.15454/1.5572338990609338E12)
Data accessibility	Repository name: Recherche Data Gouv Data identification number: Dataset 1: 10.57745/9T6Q56 Dataset 2: 10.57745/SUPRIB Dataset 3: 10.57745/EBBGE8 Dataset 4: 10.57745/GQC1L9 Direct URL to data: Dataset 1: 10.57745/9T6Q56 Dataset 2: 10.57745/SUPRIB Dataset 3: 10.57745/EBBGE8 Dataset 4: 10.57745/GQC1L9 Instructions for accessing these data: open access. Videos explaining how to use PO2 are available in Dataset 1 (named ‘Consultation of a PO2 project using PO2 Manager.mp4’ and ‘SPARQL query execution with SPO2Q.mp4’)
Related research article	

## 1 Value of the Data

• This dataset represents a broad panel of wheats grown in France exhibiting a high variability in bread making performance. It allows the comparison between wheat composition and technological quality, measured at grain, flour and bread level.
• Stakeholders in the crop industry can benefit from this dataset through the harvest reports, which can help to understand the relationship between composition and quality of use. This dataset may also be of interest to data scientists and researchers in cereal and food science.
• The dataset encompasses 35 classical technological tests, 31 detailed compositional analyses—including in-depth characterization of protein composition (glutenin and gliadin), pentosan content measurement, and fatty acid profile analysis—and 37 sensory evaluations from the French Bread baking test. These sensory evaluations provide detailed assessments of flour quality and dough behavior across key bread-making stages, including mixing, shaping, fermentation, and baking, offering comprehensive insights into both compositional and functional properties.
• Raw data sets from Alveograph® and Farinograph® have been included to allow the development of innovative criteria for wheat quality assessment.

## 2 Background

Cereals are an important resource for human and animal nutrition. With population growth, global demand is estimated at 3 billion tonnes by 2050 (i.e. a 50 % increase over 2000), of which 900 million tonnes is for common wheat (*Triticum aestivum* L.) alone. However, production is now characterised by a high degree of heterogeneity due to increasing pressures such as climate change, which increases the abiotic constraints on crops, and new sustainable agricultural practices resulting from market and societal demands. This diversity has a direct impact on the quality of production, i.e. its ability to meet certain criteria (nutritional, technological, etc.). Thus, optimising the use value of the products of agri-food systems is simultaneously becoming more critical and more complex [1].

This dataset has been developed within the framework of the French ANR EVAGRAIN research project, which aims to develop a Decision Support System (DSS) for wheat quality by integrating expert knowledge with analytical data. The project focuses on understanding the impact of minor grain components, such as pentosans and lipids, and their relationship with product quality, alongside a detailed characterization of protein composition. Each wheat sample is characterised using (1) classical analysis commonly used in the industry; (2) advanced measurements, including pentosans and lipids contents, as well as detailed protein profiling; and (3) standard French Bread Baking Test, incorporating sensory evaluations.

This data paper complements original research papers that use various approaches to predict wheat quality based on some of the measurements detailed here, by validating the proposed models [2] or allowing the establishment of new quality criteria [3].

## 3 Data Description

Data, materials and methods are stored in three datasets.

More explanations about the PO2 ontology and software ecosystem may be found in [[4], [5]].

### 3.1 *Dataset 1.* materials & methods

This dataset gathers all the information relative to the Materials and methods. It includes one file describing the origin of the feedstocks (‘Table 1 *feedstocks provenance.tab*’) as well as 11 text files, each corresponding to one method of characterization.

**Table 1:** List of all the 6 measurement tables associated with common wheat grain (CWG) extracted from the EVAGRAIN knowledge base.

Component	Step	Measurement type	name of the file including the table
common wheat grains	reception	Falling number	CWG_reception_fallingNumber_raw
common wheat grains	reception	glutenDetermination	CWG_reception_glutenDetermination_raw
common wheat grains	reception	Hardness	CWG_reception_hardness_raw
common wheat grains	reception	NIRSGrainContentAnalysis	CWG_reception_NIRSGrainContentAnalysis_raw
common wheat grains	reception	specificWeight	CWG_reception_specificWeight_raw
common wheat grains	reception	thousandKernelsWeight	CWG_reception_thousandKernelsWeight_raw

In Table 1 *(‘feedstock*s *provenance’*), the third column *sampleCode* corresponds to the unique reference of the wheat samples harvested, relative to production routes (or itinerary), while the columns five to nine contain metadata describing the variety and technological class of the wheat samples and their geographical origin.

The dataset also includes the SPARQL query used to generate Table 1 from the online tools SPO2Q. Queries and table have the same name, only the extension differs by replacing the string ‘*tsv*’ by ‘*sparql*’: ‘Table 1 *feedstocks provenance.sparql’* is the name of the query associated with table *‘*Table 1 *feedstocks provenance.tsv’.* Furthermore, the dataset also includes two videos: one demonstrating the execution of SPARQL queries stored in **datasets 1** and **2** (‘*SPARQL query execution with SPO2Q*’), and another explaining the installation of PO2 Manager and how to access the entire experimental project using this desktop application (‘*Consultation of a PO2 project using PO2 Manager*‘).

The three text files (1-Plant Material.txt, 2-Wheat Grain and Flour Classical Characterization.txt, 3-Advanced Characterization of Flour Composition.txt) describe the materials and methods presented in the following section Experimental design, Materials, and Methods.

### 3.2 *Dataset 2.* Raw and calculated data

**Fig. 1:**
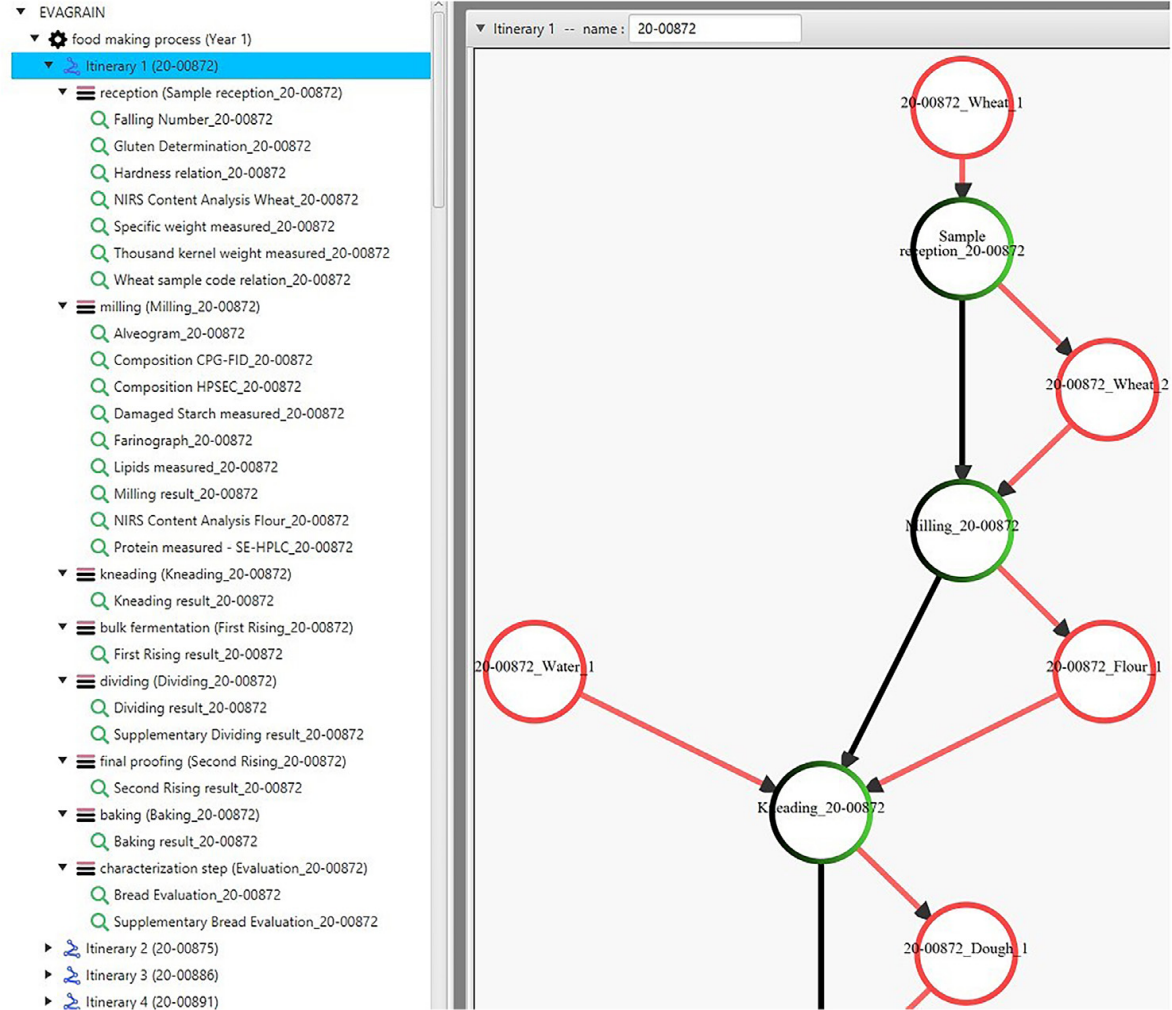
Structuration of the knowledge base including transformation steps and associated measurements.

This data is available under two accessible forms: a single CSV file grouping all the measurements for each sample, and a knowledge graph structured by an ontology dedicated to transformation processes [5]. The latter allows complex queries and ensures data transparency, by keeping track of the methods and materials used for each measurement. Fig. 1 presents the data organisation in the knowledge base for an excerpt corresponding to wheat sample 20-00872. This is a screenshot from the PO2Manager tool, which permits navigation through the knowledge graph and data visualisation. The right side is an excerpt from the graphical representation of the biomass transformation itinerary: components are represented by red circles, while transformation steps are shown as black circles. Green colouring within the black circles indicates that measurements were taken on output components of the given step. The left side lists all observations, including measurements associated with the steps. For example, Falling number and Gluten determination are observations associated with the reception step.

The data stored in the knowledge base were extracted using SPARQL queries, resulting in 29 tables that correspond to measurements taken on four components: common wheat grains, wheat flour, dough and bread (see Tables 1, 2, Table 3, Table 4 below).

**Table 2:** List of all the 13 measurement tables associated with wheat flour (WF) extracted from the EVAGRAIN knowledge base.

Component	Step	Measurement type	name of the file including the table
wheat flour	milling	Alveograph	WF_milling_alveograph_raw
wheat flour	milling	Polysaccharide composition	WF_milling_Polysaccharide composition1_calc
wheat flour	milling	Polysaccharide composition	WF_milling_Polysaccharide composition2_calc
wheat flour	milling	Polysaccharide composition	WF_milling_Polysaccharide composition3_calc
wheat flour	milling	Polysaccharide PhysicoChemical Characteristicsc	WF_milling_polysaccharidePhysicoChemicalCharacteristics_calc
wheat flour	milling	damagedStarch	WF_milling_damagedStarch_raw
wheat flour	milling	Farinograph	WF_milling_farinograph_raw
wheat flour	milling	Lipids Measured	WF_milling_lipidsMeasured_part1_calc
wheat flour	milling	Lipids Measured	WF_milling_lipidsMeasured_part2_calc
wheat flour	milling	Flour Moisture Content	WF_milling_FlourMoistureContent_calc
wheat flour	milling	millingResults	WF_milling_millingResults_calc
wheat flour	milling	proteinsMeasured	WF_proteinsMeasured_SE_HPSEC_calc

**Table 3:** List of all the 6 measurement tables associated with dough (D) extracted from the EVAGRAIN knowledge base.

Component	Step	Measurement type	name of the file including the table
dough	kneading	breadMakingTest_kneadingResults	D_kneading_breadMakingTest_kneadingResults_raw
dough	bulkFermentation	breadMakingTest_firstRising	D_bulkFermentation_breadMakingTest_firstRising_raw
dough	dividing	breadMakingTest_dividingResults	D_dividing_breadMakingTest_dividingResults_raw
dough	dividing	breadMakingTest_DividingSupplementary	D_dividing_breadMakingTest_DividingSupplementary_raw
dough	finalProofing	breadMakingTest_secondRising	D_finalProofing_breadMakingTest_secondRising_raw
dough	baking	breadMakingTest_bakingResults	D_baking_breadMakingTest_bakingResults_raw

**Table 4:** List of all the 4 measurement tables associated with bread (B) extracted from the EVAGRAIN knowledge base.

Component	Step	Measurement type	name of the file including the table
bread	characterisation	breadEvaluation_part1	B_characterisation_breadEvaluation_raw_part1
bread	characterisation	breadEvaluation_part2	B_characterisation_breadEvaluation_raw_part2
bread	characterisation	breadEvaluationGlobal	B_characterisation_breadEvaluation_calc
bread	characterisation	breadEvaluation_supplementaryBreadEvaluation	B_characterisation_breadEvaluation_supplementaryBreadEvaluation

The file names associated with the tables have the following syntax: *component_step_measurement_type[raw/calc],* where “*raw”* indicates that data were directly retrieved from the characterization material, and “*calc” signifies* that the data were computed using the formulas presented in the *Experimental design, material and methods section* below.

**Fig. 2:**
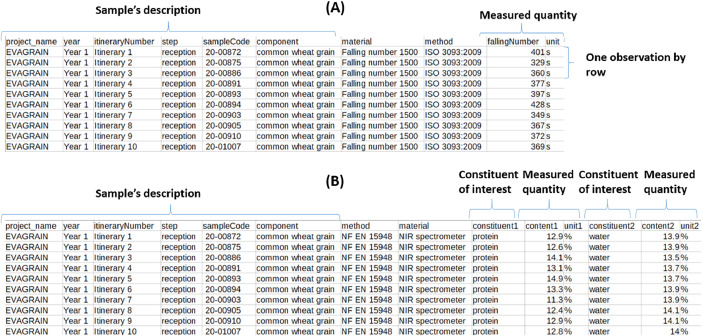
Two examples of measurement tables: (A) the measured value is associated with the component: in the example, the falling number is associated with the common wheat grain sample; (B) measured values are associated with constituents of the component: in the example, the content of constituent protein (resp. water) is associated with the common wheat grain sample.

The set of 29 measurement tables is structured in the same format as shown in Fig. 2. The sample description includes the following information: harvest year (year 1 or year 2), itinerary number (a unique identifier in the knowledge base), the step in which the component is either input or output, the sample code (a unique identifier for the sample in the research project), and the component being characterised. As shown in Fig. 2, two types of tables are possible: (A) characterisation of the component, (B) characterisation of one or more constituents of the component. In case of analytical repetitions, average and standard deviation are expressed using the format [[average; standard deviation]] in the cell.

### 3.3 *Dataset 3.* Queries associated with measurement tables

This dataset consists of 29 SPARQL queries executed to generate the measurement tables included in the files belonging to dataset 2. The queries shared the same names differing only by their extension. For example, *CWG_reception_fallingNumber_raw.sparql* contains the SPARQL query used to generate the table found in *CWG_reception_fallingNumber_raw.tsv*.

### 3.4 *Dataset 4.* Raw datasets from alveograph and farinograph

Dataset 4 contains raw data files for alveograph and farinograph measurements, available in .txt or .xls formats. All files are organized into folders by harvest year, with each sample stored in its own sub-folder. For the alveograph, filenames correspond to the sample code. For example, *20-00872.txt* is the raw data file for sample 20-00872. For the farinograph, filenames include the sample code and consists of 4 groups of numbers. For example, *‘865 22 20 00886.xls’* is the raw data file for sample 20-00886.

## 4 Experimental Design, Materials and Methods

### 4.1 Plant material

#### 4.1.1 Origin

A total of 290 wheat samples, harvested in 2020 and 2022, were used to cover a wide range of end-use quality. These samples represent 99 different varieties cultivated across 39 locations in France, identified by commune, department, and region, to capture diverse agro-pedo-climatic conditions. The samples were provided by Arvalis^1^ (Boigneville, France), Limagrain^2^ (Riom, France), and Axiane Meunerie^3^. (Gallardon, France). Additionally, each sample was categorized by the technological class defined by CTPS (Comité Technique Permanent de la Sélection des Plantes Cultivées) for French cultivars.

#### 4.1.2 Geographic diversity

**Fig. 3:**
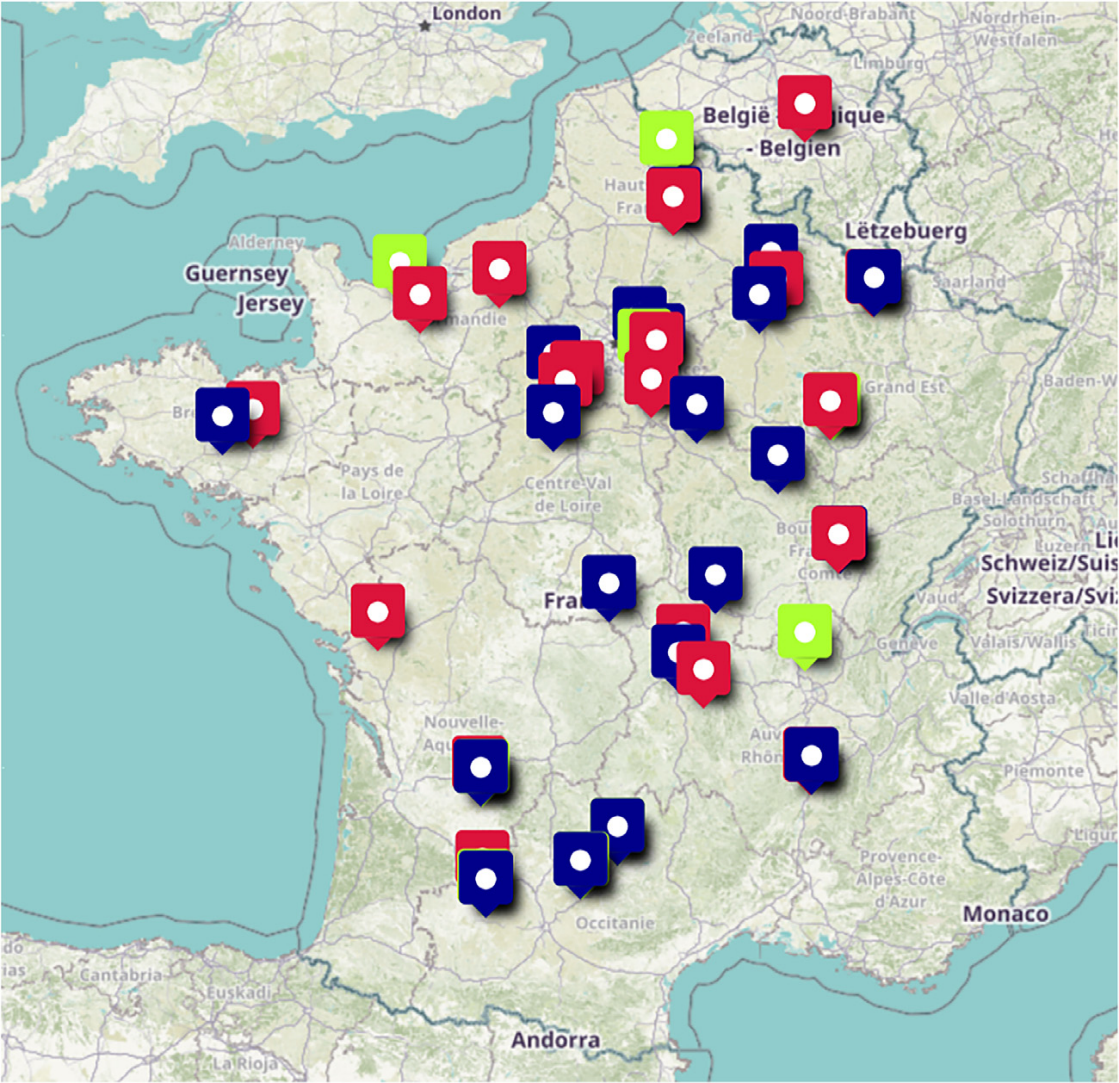
Localization in France and Belgium of each wheat plot grown during the study. Colour represents the harvest year. Green: 2020; Blue: 2021; Red: 2022.

The diversity of the selected plots across France and Belgium is illustrated in Fig. 3. Color coding indicates the harvest year: green for 2020, blue for 2021, and red for 2022.

#### 4.1.3 Milling process

The harvested grains were milled into white flour with an average ash content of 0.55 %, corresponding to Euro 550 flour type and American all-purpose flour (predominantly grain endosperm). Milling was performed using an experimental mill (MCKA, Bühler, Switzerland) in batches of 10 kg. The milling process was characterized by its dry matter extraction rate and its yields in tail-end flour, bran, wheat middlings, break flour, and reduction flour.

#### 4.1.4 Storage conditions

The milled flours were stored at room temperature for 20 days post-milling and subsequently frozen at −20 °C until analysis. Before analysis, samples were thawed overnight at room temperature**.**

### 4.2 Wheat grain and flour classical characterization

#### 4.2.1 Analysis

The following characteristics were measured.

##### 4.2.1.1 Grain properties

• Protein and Water Content: Determined via near-infrared spectroscopy (NIRS) using protocol NF EN 15948:2020.
• Grain Hardness: Assessed using the Particle Size Index (PSI), calibrated with NIRS.
• Specific Weight: Measured according to NF EN ISO 7971-3.
• Thousand Kernel Weight: Determined using NF EN ISO 520**.**

##### 4.2.1.2 Starch and gluten properties

• Hagberg Falling Number: Measured with a Falling Number 1500 following ISO 3093:2009.
• Damaged Starch (SD): Quantified using SDMatic® (Chopin, France) via iodine absorption with amperometric detection, expressed in Chopin Units corrected for protein and moisture content (UCDc).
• Gluten Parameters: Wet Gluten, Dry Gluten, and Gluten Index were determined using Glutomatic®, following NF EN ISO 21415-2.

##### 4.2.1.3 Dough rheological properties

• Alveograph Analysis: Characteristics such as tenacity (P), extensibility (L), the tenacity-to-extensibility ratio (P/L), baking strength (W), and elasticity index (Ie) were assessed per NF EN ISO 27971 (Chopin, France).
• Mixolab Analysis: Water absorption, development time, stability, and weakening degree were measured using Mixolab® (Chopin, France), following NF V03–765. Water absorption corresponds to the percentage of water required for the dough to reach a torque of 1.1 Nm, equivalent to the Farinograph® determination**.**

#### 4.2.2 Wheat quality: the bread making test

Wheat quality was evaluated following the classical French Bread Baking Test (AFNOR standard NF V03–716), which is widely used in France to assess the baking value of common wheat. This test covers the key stages of the breadmaking process, from kneading to the final baked bread, including crumb and crust evaluation. For each stage, several sensory observations were recorded:

• Kneading: After mixing the ingredients for common French bread, six dough attributes were evaluated: smoothing, stickiness, consistency, extensibility, elasticity, and slackening.
• First Rising: The dough was left to rise for the first time. Dough slackening was measured.
• Dividing: After the first rise, the dough was divided and shaped into future loaves. Four dough attributes were evaluated: lengthening, tearing, elasticity and stickiness.
• Second Rising: The shaped dough pieces were left to rise a second time. Two attributes were measured: rising level and tearing.
• Baking: The dough pieces were baked. Before being placed in the oven, two dough attributes were measured: stickiness and free-standing.
• Bread Analysis: The final product was evaluated through three criteria: the bread itself, the crumb and the volume. Bread attributes include section, colour, thickness, crispiness, and knife cut development, regularity, and tearing. Crumb attributes include colour, plasticity, stickiness, regularity, and odour.

Sensory attributes were evaluated on a rated scale with a maximum of seven values ranging from Insufficiency to Excess (−1 < −4 < -7 < 10 < 7 < 4 < 1), with the reference value for a standard French bread making process set as the central value of 10. Some attributes, such as stickiness, only use one-half of the sensory scale, allowing evaluation from standard to excess.

The results are presented in a specific evaluation grid, from which three scores are calculated as a weighted sum on a scale from 0 to 100: one for the dough, one for the bread aspect and one for the crumb. The sum of these three scores yields the overall score of the baking test, which is widely used in the wheat production sector to distinguish between gold standard and defective quality products.

### 4.3 Advanced characterization of flour composition

#### 4.3.1 Polysaccharide analysis

##### 4.3.1.1 Alcohol insoluble materials (AIMs) extraction

Flour samples (2 g) were extracted into AIMs using an ASE® 350 accelerated solvent extractor (Thermo, USA) with ethanol/water (80:20, v/v). Extraction conditions were set at 100 °C, with a flow rate of 2 mL/min, a static time of 20 min, a rinse volume of 150 %, and a 30-second nitrogen purge. AIMs were dried at 40 °C for 3 h and then under vacuum overnight over P₂O₅. Afterward, the dried AIMs were finely ground using an IKA Tube Mill 100 control with a 30-second grinding duration. The dried and ground AIMs were weighed, and the mass was recorded to calculate AIM yield. The AIMs were stored at room temperature for further analysis.

##### 4.3.1.2 Aqueous extracts

AIMs (1 g) were suspended in 4 mL of ultrapure water in 15 mL Falcon tubes, vortexed for 10 ss, and shaken overnight (16 h) at 1700 rpm at 40 °C using a Heidolph Multi Reax shaker. After incubation, the tubes were vortexed again, centrifuged at 2200 g for 30 mins at 25 °C, and the supernatant (2 mL) was transferred to 2 mL Eppendorf tubes for further analysis.

##### 4.3.1.3 Polysaccharide composition and analysis

The monosaccharide composition of AIMs and aqueous extracts was determined by gas chromatography (GC) following acid hydrolysis [6]. Total polysaccharide content in AIMs was assessed by hydrolyzing 5 mg of AIMs in 1 M H₂SO₄ at 100 °C for 2 h. Similarly, water-extractable polysaccharides were quantified by hydrolyzing 0.2 mL of the supernatant under identical conditions. Inositol was used as an internal standard. Hydrolyzed monosaccharides were derivatized to alditol acetates and analyzed by gas-liquid chromatography (GLC) using a TG-225MS column (Thermo, 30 m × 0.25 mm ID × 0.25 µm film thickness) on a Trace GC Ultra system (Thermo Fisher Scientific). The column temperature was maintained at 205 °C, and hydrogen was the carrier gas. Data were analyzed in triplicate, and results were expressed as mean values ± standard deviations. Total arabinose content (Ara) and total xylose content (Xyl) were obtained from AIMs, while water-extractable arabinose content (Ara.WE) and Water-extractable xylose content (Xyl.WE) were obtained from aqueous extracts.

##### 4.3.1.4 Arabinoxylan quantification and fractionation

Total arabinoxylan content (TOT-AX), water-extractable arabinoxylan (TOT-AX.WE), and water-unextractable arabinoxylan (TOT-AX.WU) fractions were calculated as follows:

• TOT-AX = Ara_AX + Xyl
• TOT-AX.WE = Ara_AX.WE + Xyl.WE
• TOT-AX.WU = Ara_AX.WU + Xyl.WU (with Ara_AX.WU=Ara_AX-Ara_AX.WE and Xyl.WU=Xyl-Xyl.WE)

Arabinose from total arabinoxylan (Ara_AX) and arabinose from water-extractable arabinoxylans (Ara_AX.WE) values were corrected for contributions from arabinogalactan-proteins (AGPs) based on an Ara_AGP:Gal_AGP ratio of 0.7, assuming all AGP-derived arabinose was water-soluble. Arabinose to xylose ratios A/X were calculated to assess the degree of substitution in arabinoxylans.

##### 4.3.1.5 High-performance size-exclusion chromatography (HPSEC) analysis

Aqueous extracts (1.8 mL) were incubated with 3 units of thermostable α-amylase (Megazyme, Bacillus sp., E-BSTAA) overnight at 30 °C to remove starch. The treated solutions were filtered through a 0.45 µm membrane and analyzed via HPSEC using a Malvern Panalytical OMNISEC RESOLVE-REVEAL system. The column setup consisted of a Viscotek AGuard precolumn and a Viscotek A4000 column, maintained at 35 °C. The mobile phase was 50 mM sodium nitrate, flowing at 0.7 mL/min. Detection was performed using a differential refractometer, multi-angle laser light scattering (MALLS, λ = 660 nm), and a viscometer. Calibration involved a pullulan standard (Mw = 40,611 Da, dn/dc = 0.147 mL/g), and analysis used OMNISEC v11.32 software. For TOT-AX.WE, dn/dc was set to 0.146 mL/g [7].

Water-extractable arabinoxylan concentrations (WEAX_HPSEC_) were calculated using the refractive index signal, correlating with TOT-AX.WE (R² = 0.83). Intrinsic viscosity (IV.AX) of TOT-AX.WE was derived from the viscometer's differential pressure signal as described in [2,8].

#### 4.3.2 Lipid analysis

##### 4.3.2.1 Non-starch lipid extraction

Non-starch lipids were extracted from 500 mg of flour using hexane/propanol (3:2, v/v) with an ASE® 350 system at room temperature. The system operated with a 3-minute flow time, 100 % rinse volume, and a 30-second nitrogen purge. Lipid extracts were evaporated to dryness under reduced pressure at 45 °C using a Genevac system.

##### 4.3.2.2 Fatty acid composition

Extracted lipids were transmethylated using 4 mL of 2 % H₂SO₄ in methanol for 2 h at 60 °C, with intermittent shaking during the first hour [9]. After cooling, 1.5 mL cyclohexane and 2 mL ultrapure water were added to the reaction mixture, vortexed briefly, and the upper phase was collected after overnight storage at 4 °C. Fatty acid methyl esters were quantified using gas-liquid chromatography with a DB225 column (30 m × 0.25 mm ID × 0.25 µm film thickness) at 250 °C and hydrogen as the carrier gas.

The total fatty acids measured were as follows: total palmitic acid C16 content (C16.TOT), total stearic acid C18 content (C18.TOT), total vaccenic acid C18:1n-7 content (C181n7.TOT), total oleic acid C18:1n-9 content (C181n9.TOT), total linoleic acid C18:2n-6 content (C182n6.TOT) and total alpha-linolenic acid C18:3n-3 content (C183n3.TOT).

The Non-Starch fatty acids measured were as follows: non-starch palmitic acid C16 content (C16.NS), non-starch vaccenic acid C18:1n-7 content (C181n7.TOT), non-starch linoleic acid C18:2n-6 content (C182n6.NS) and non-starch alpha-linolenic acid C18:3n-3 content (C183n3.NS).

The amount of fatty acids bound to starch (S) was calculated by subtracting the amount of non-starch (NS) fatty acids as detected in the hexane/propanol extracts from the total fatty acid content (TOT) measured in the flour.

#### 4.3.3 Protein analysis

##### 4.3.3.1 Sequential extraction of gluten proteins

Flour samples (160 mg) were subjected to sequential extraction with 20 mL of 0.1 M sodium phosphate buffer (pH 6.9) containing 1 % SDS. The first extraction was performed at 60 °C for 80 min with shaking (Heidolph Reax 2, setting 5), followed by centrifugation at 18,000 rpm at 25 °C for 30 min. The resulting pellet was re-extracted with 5 mL of the buffer and subjected to 180 s of sonication at 30 % power.

##### 4.3.3.2 HPSEC analysis of gluten fractions

Supernatants were analyzed by HPSEC using a TSKgel® G4000-SWXL column and 0.1 M phosphate buffer containing 0.1 % SDS as the mobile phase. Detection was performed at 214 nm considering a specific extinction coefficient of 18.51 L/g/cm for the wheat protein. Calibration of the column was carried out using molecular weight standards from the Agilent PSS-PROKIT. Total glutenin (GluT), soluble glutenin (GluS), insoluble glutenin (GluI), and gliadin (Gli) fractions were quantified from chromatograms as described in [10].

## Limitations

None

## Ethics Statement

The authors have read and follow the ethical requirements for publication in Data in Brief and confirm that the current work does not involve human subjects, animal experiments, or any data collected from social media platforms.

## CRediT Author Statement

**Mélanie Munch:** Data curation, Writing - Original draft, Writing - Review & Editing. **Laura Rezette**: Methodology, Investigation, Resource, Writing - Original draft, Writing - Review & Editing. **Patrice Buche**: Software, Data curation, Writing - Original draft, Writing - Review & Editing. **Baptiste Chambrey**: Resource. **Catherine Deborde**: Writing - Review & Editing. **Stéphane Dervaux**: Software. **Sonia Geoffroy**: Conceptualization, Methodology, Investigation, Resource. **Kamal Kansou**: Conceptualization, Methodology, Data curation. **Sophie Le Gall**: Conceptualization, Methodology, Investigation, Resource, Supervision. **Laurent Linossier**: Conceptualization, Methodology, Investigation, Resource. **Benoit Meleard**: Conceptualization, Methodology, Investigation, Resource. **Luc Menut**: Software, Data curation. **Marie-Hélène Morel**: Conceptualization, Methodology, Investigation, Resource. **Magalie Weber**: Data curation. **Luc Saulnier**: Conceptualization, Methodology, Funding acquisition, Supervision.

## Data Availability

entrepot.recherche.data.gouv.frRaw and Calculated Analytical Data (Original data). entrepot.recherche.data.gouv.frMaterials & Methods (Original data). entrepot.recherche.data.gouv.frFarinograph and Alveograph Curves (Original data). entrepot.recherche.data.gouv.frRaw and Calculated Analytical Data SPARQL Queries (Original data).

## Footnotes

1 https://www.arvalis.fr/technical-institute.

2 https://www.limagrain.com/en.

3 https://www.axiane.com/en
